# Azithromycin-Induced Liver Injury, a Case Report

**DOI:** 10.51894/001c.164424

**Published:** 2026-08-01

**Authors:** Emily Andresan, Zachary Kam, Saleem Mahmoud Halablab, Hadi Mansour, Fouad Alzaroui, Aakash Srikanth, Aayush Mittal

**Affiliations:** 1 Henry Ford Detroit

## 59

### Introduction

Drug-induced liver injury (DILI) is a challenging diagnosis and can present with a cholestatic pattern. Although azithromycin is widely used and typically well tolerated, it is a rare cause of idiosyncratic cholestatic DILI.

### Case DESCRIPTION

We describe an 81-year-old man with COPD, coronary artery disease, and chronic alcohol use who developed progressive jaundice and fatigue shortly after receiving two courses of azithromycin for an upper respiratory infection within the preceding month. Initial evaluation at an outside hospital showed a predominantly cholestatic liver injury pattern and anemia in the setting of melena, prompting transfer to a tertiary center. Imaging did not demonstrate biliary obstruction, and an extensive workup for alternative etiologies (including viral and autoimmune causes) was unrevealing. He was managed supportively with medication review, continued ursodiol, and close laboratory monitoring. Liver chemistries began to improve, and he was discharged with follow-up.

### Discussion

The timing of exposure, cholestatic laboratory profile, and exclusion of competing diagnoses supported azithromycin-induced DILI; RUCAM scoring indicated a “probable” causal relationship.

### Conclusion

This case underscores the importance of a careful antibiotic history when evaluating cholestatic hepatitis, particularly in older adults, to avoid delays in diagnosis and prevent progression to more severe cholestatic complications

